# Role of continuous glucose monitoring in diabetic patients at high cardiovascular risk: an expert-based multidisciplinary Delphi consensus

**DOI:** 10.1186/s12933-022-01598-2

**Published:** 2022-08-27

**Authors:** Carlo Di Mario, Stefano Genovese, Gaetano A. Lanza, Edoardo Mannucci, Giancarlo Marenzi, Edoardo Sciatti, Dario Pitocco, the Expert Panel Group, Angelo Avogaro, Federico Bertuzzi, Enzo Bonora, Claudio Borghi, Raffaella Buzzetti, Stefano Carugo, Davide Capodanno, Agostino Consoli, Antonio Conti, Rossella Danesi, Paolo Bartolo, Gaetano Maria De Ferrari, Stefano Favale, Carlo Giorda, Francesco Giorgino, Angela Girelli, Paolo Golino, Francesco Grigioni, Ciro Indolfi, Concetta Irace, Elisabetta Lovati, Ada Maffettone, Maria Masulli, Fabrizio G Oliva, Luigi Oltrona Visconti, Emanuela Orsi, Uberto Pagotto, Leonardo Paloscia, Gianfranco Parati, Pasquale Perrone, Gianfranco Piccirillo, Paolo Pozzilli, Giuseppe Pugliese, Francesco Purrello, Flavio Ribichini, Andrea Rubboli, Michele Senni, Roberto Trevisan, Claudio Tubili, Massimo Uguccioni

**Affiliations:** 1 Cardiology Unit, AOU Careggi and University of Florence, Florence, Italy; 2 Diabetes, Endocrine and Metabolic Diseases Unit, Centro Cardiologico Monzino IRCCS, Milan, Italy; 3 Noninvasive Diagnostic Cardiology Unit, Fondazione Policlinico Universitario A. Gemelli IRCCS, Università Cattolica del Sacro Cuore, Rome, Italy; 4 Diabetology Unit, AOU Careggi and University of Florence, Florence, Italy; 5 Intensive Cardiac Care Unit, Centro Cardiologico Monzino IRCCS, Milan, Italy; 6 Cardiology Unit 1, ASST Papa Giovanni XXIII, Bergamo, Italy; 7 Diabetology Unit, Fondazione Policlinico Universitario A. Gemelli IRCCS, Università Cattolica del Sacro Cuore, Rome, Italy

**Keywords:** Delphi method, Continuous glucose monitoring, Cardiovascular outcome, Time in range, Glycaemic variability, Glucometrics

## Abstract

**Background:**

Continuous glucose monitoring (CGM) shows in more detail the glycaemic pattern of diabetic subjects and provides several new parameters (“glucometrics”) to assess patients’ glycaemia and consensually guide treatment. A better control of glucose levels might result in improvement of clinical outcome and reduce disease complications. This study aimed to gather an expert consensus on the clinical and prognostic use of CGM in diabetic patients at high cardiovascular risk or with heart disease.

**Methods:**

A list of 22 statements concerning type of patients who can benefit from CGM, prognostic impact of CGM in diabetic patients with heart disease, CGM use during acute cardiovascular events and educational issues of CGM were developed. Using a two-round Delphi methodology, the survey was distributed online to 42 Italian experts (21 diabetologists and 21 cardiologists) who rated their level of agreement with each statement on a 5-point Likert scale. Consensus was predefined as more than 66% of the panel agreeing/disagreeing with any given statement.

**Results:**

Forty experts (95%) answered the survey. Every statement achieved a positive consensus. In particular, the panel expressed the feeling that CGM can be prognostically relevant for every diabetic patient (70%) and that is clinically useful also in the management of those with type 2 diabetes not treated with insulin (87.5%). The assessment of time in range (TIR)*,* glycaemic variability (GV) and hypoglycaemic/hyperglycaemic episodes were considered relevant in the management of diabetic patients with heart disease (92.5% for TIR, 95% for GV, 97.5% for time spent in hypoglycaemia) and can improve the prognosis of those with ischaemic heart disease (100% for hypoglycaemia, 90% for hyperglycaemia) or with heart failure (87.5% for hypoglycaemia, 85% for TIR, 87.5% for GV). The experts retained that CGM can be used and can impact the short- and long-term prognosis during an acute cardiovascular event. Lastly, CGM has a recognized educational role for diabetic subjects.

**Conclusions:**

According to this Delphi consensus, the clinical and prognostic use of CGM in diabetic patients at high cardiovascular risk is promising and deserves dedicated studies to confirm the experts’ feelings.

## Background

Self-monitoring of blood glucose (SMBG) has until now been the most widely used method by patients with diabetes to assess their own glycaemia and guide diabetes treatment. On the other hand, glycated haemoglobin A1c (HbA1c) is the primary tool for assessing glycaemic control and has a strong predictive value for diabetes complications [1–6]. However, both these established methods of diabetes assessment present well-known limitations. SMBG only provides data at a single point, is time consuming, inconvenient, and painful, often leading to poor adherence [7]. HbA1c only reflects the average glycaemia of the last 3 months, is not reliable in the presence of some pathological conditions such as anaemia and does not give information on blood glucose fluctuations caused by food intake, physical activity, medication or any other physical or emotional stress.

In the last decade techniques have been developed that allow continuous monitoring of blood glucose levels, which provide the unique opportunity to analyse in detail, even for several days, the glycaemic pattern (i.e., glucose levels and their variations) of diabetic patients [8].

Continuous glucose monitoring (CGM) devices use a fixed sensor with a subcutaneous glucose-oxidase platinum electrode that measures glucose concentrations in the interstitial fluids [9]. They either continuously track the glucose concentration providing real-time data, namely real-time CGM (rtCGM), or show continuous measurements intermittently scanned “on-demand”, namely intermittently scanned CGM (isCGM) or flash glucose monitoring (FGM) [10].

CGM provides several new parameters (“glucometrics”) that may better reflect patients’ glycaemic values and consensually improve their treatment. Importantly, some data suggest that the management of diabetic patients by CGM might also improve clinical outcome and reduce the risk of complications [10]. The most important of the glucometrics derived from CGM include the time in range (TIR), defined as the time with glycaemia fitting among two cut-offs of 70 and 180 g/dL [11], and glycaemic variability (GV), which reflects the amplitude and the frequency of glycaemic fluctuations [12, 13]. CGM derived glucometrics overcome the main issues related to SMBG and HBA1c providing novel, easy-to-get and unpainful data about glucose fluctuations, including the detection of relevant hyperglycaemic and, even more, hypoglycaemic events [13], which have consistently been associated with a worse clinical outcome in diabetic patients [14].

Taken together, these considerations let a recent consensus of diabetologists acknowledge the obsolescence of SMBG and limitations of HbA1c, highlighting the need of using new tools and glucometrics to improve glycaemic control and therapeutic management [15]. Moreover, CGM has been gradually improved in terms of easiness to use, accuracy, reliability, and cost effectiveness. Accordingly, the American Diabetes Association (ADA) has recently recommended its use for the management of both patients with type 1 (T1DM) and type 2 (T2DM) diabetes mellitus treated with multiple daily insulin injections [16].

Notably, the recent publication of randomized clinical trials (RCT) showing improved cardiovascular (CV) outcome in diabetic patients treated with some new anti-hyperglycaemic agents [17] has raised cardiologists’ interest towards appropriate treatment of diabetes and stimulated new relations between cardiologists and diabetologists. However, it seems now necessary to extend to the cardiologists the knowledge of CGM and new glucometrics, letting them provide this diagnostic option to achieve a better glycaemic control in their diabetic patients with high CV risk or overt heart disease. However, several uncertainties and lack of evidence exist in this field.

Considering this background, the purpose of this study was to perform a Delphi survey among a panel of Italian diabetologists and cardiologists to gather an expert consensus on the use of CGM in diabetic patients at high CV risk or with a history of CV events.

## Methods

The Delphi method is a structured technique aimed at obtaining, by repeated rounds of questionnaires, a consensus opinion from a panel of experts in areas where evidence is scarce, and opinion is important [18–20]. In the present study, the consensus process consisted of a double-step web-based Delphi method, which took place between May and September 2021.

The online survey was developed by a panel of six physicians (three couples of diabetologists-cardiologists from three Italian excellence centres), identified here as key opinion leaders (KOLs) in their respective field in Italy. The KOLs virtually met to fully analyse the published literature and discuss the unmet needs about the topic. Hence, they identified 22 statements, which were in serious need of clarification and debate, all focused on CGM use: type of patients who can benefit from CGM, prognostic impact of CGM in diabetic patients with heart disease, CGM use during acute CV events, educational issues of CGM (Table 1). Notably, at the time of the survey no retrospective (Holter-like) CGM system was commercially available in Italy; for this reason, the questions were referred to real-time CGM and FGM only, unless otherwise specified.

**Table 1:** The survey

Statement 1: Type of patients who can benefit from continuous glucose monitoring (*flash* and *classic CGM*)	
1.1 1.2 1.3 1.4 1.5 1.6	Based on the data available to date, continuous glucose monitoring can represent a valid prognostic tool in every person affected by diabetes mellitus Continuous glucose monitoring is prognostically useful in patients with type 1 diabetes and in patients with type 2 diabetes on multiple daily insulin injections Continuous glucose monitoring in type 2 diabetes treated with basal insulin and/or oral hypoglycaemic agents and/or other injecting drugs is useful in clinical characterization of the patient and in making treatment decisions (e.g., to monitor change of therapy) Continuous glucose monitoring is particularly useful in patients with physical limitations that prevent blood glucose measurement with the traditional capillary test (e.g., severe arthritis, Parkinson's disease, other) Continuous glucose monitoring is useful in patients in whom a rapid improvement in blood glucose control is clinically indicated (e.g., post-myocardial infarction, pre-/post-surgery, sepsis, acute respiratory failure, acute renal failure) Continuous glucose monitoring facilitates telemonitoring in elderly patients
Statement 2: Prognostic impact of continuous glucose monitoring in diabetic patients affected by heart disease	
2.1 2.2 2.3 2.4 2.5 2.6 2.7 2.8 2.9	The time-in-range (percentage of monitoring time with blood glucose values ​​between 70 and 180 mg/dL) is a parameter that provides more complete and detailed information than HbA1c in the clinical evaluation of people with diabetes The reduction in hypoglycaemic episodes improves the prognosis of diabetic patients with ischemic heart disease The reduction of hyperglycaemic episodes improves the prognosis of diabetic patients with ischemic heart disease Time-in-range is an important element for therapeutic optimization in the diabetic patient with heart disease The time spent in hypoglycaemia is an important element for therapeutic optimization in the diabetic patient with heart disease Glycaemic variability is an important element for therapeutic optimization in the diabetic patient with heart disease Time-in-range has a prognostic role in the diabetic patient with heart failure Time spent in hypoglycaemia has a prognostic role in the diabetic patient with heart failure Glycaemic variability has a prognostic role in the diabetic patient with heart failure
Statement 3: Use during acute cardiovascular events	
3.1 3.2 3.3 3.4	Continuous glucose monitoring can be easily used in coronary care unit/intensive care unit Reduction in hypoglycaemic episodes improves short-term prognosis during an acute cardiovascular event Reduction in hyperglycaemic episodes improves short-term prognosis in patients with an acute cardiovascular event The optimization of time-in-range and glycaemic variability and the reduction of time in hypoglycaemia are associated with an improvement in the long-term prognosis in patients with an acute cardiovascular event
Statement 4: Insulin/hypoglycaemia education	
4.1 4.2 4.3	Continuous glucose monitoring helps patients with diabetes mellitus improve the perception of their disease Continuous glycaemic monitoring can be helpful in reducing hypoglycaemic episodes The possibility to use alarms, now available in all interstitial glucose sensors, helps prevent hypoglycaemias

Once developed, the survey was evaluated by 6 external validators chosen by the panel to test its understandability and clarity. Following this, the questionnaire was distributed to 21 couples of expert diabetologists-cardiologists via an online survey platform with anonymized results. The experts were clinicians with solid experience in their respective field, selected throughout the country among Unit directors, University professors and national and international Scientific Societies members, so that the whole country was homogeneously represented [19].

Diabetologists and cardiologists were asked to express their level of agreement or disagreement with each statement on a 5-point Likert scale, scored as follows: 1, extremely disagree; 2, disagree; 3, agree; 4, mostly agree; and 5, extremely agree. All answers were categorized into two categories: for the purpose of this study, ‘‘extremely disagree’’ and ‘‘disagree’’ were categorized into category ‘‘Negative Consensus’’; ‘‘agree’’, ‘‘mostly agree’’ and ‘‘extremely agree’’ were categorized into ‘‘Positive Consensus’’. A cut-off of 66% of agreement/disagreement was chosen a priori to represent positive or negative consensus, respectively. No consensus was reached when < 66% of the answers fell in the same category [19, 20]. There was no need to re-rate any statement since every declaration reached consensus at the first round.

**Fig. 1:**
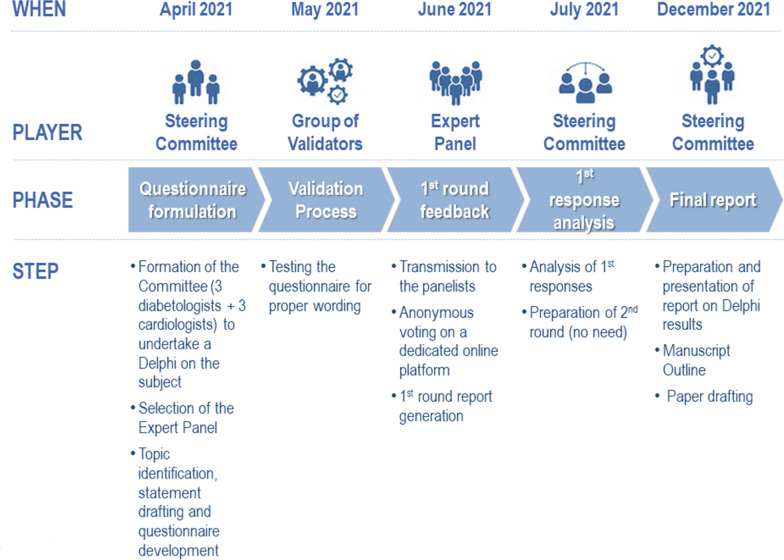
Description of the Delphi process

The Delphi process is resumed in Fig. 1.

Descriptive statistics were used to summarize the results. A sub-analysis of the results according to the area of expertise (diabetology vs cardiology) was also provided.

The study is based on a survey that does not involve the participation of human subjects nor patient data management and does not aim to modify the current clinical practice of participants. Consequently, this study did not require ethical approval. All experts involved in the Delphi survey were informed of the study’s objectives and the possibility of publishing the results in a peer-reviewed article. The participation was voluntary. They expressed their consent to participate in the survey after logging into the secure online survey platform via credentials, by actively clicking on the appropriate box.

## Results

**Fig. 2:**
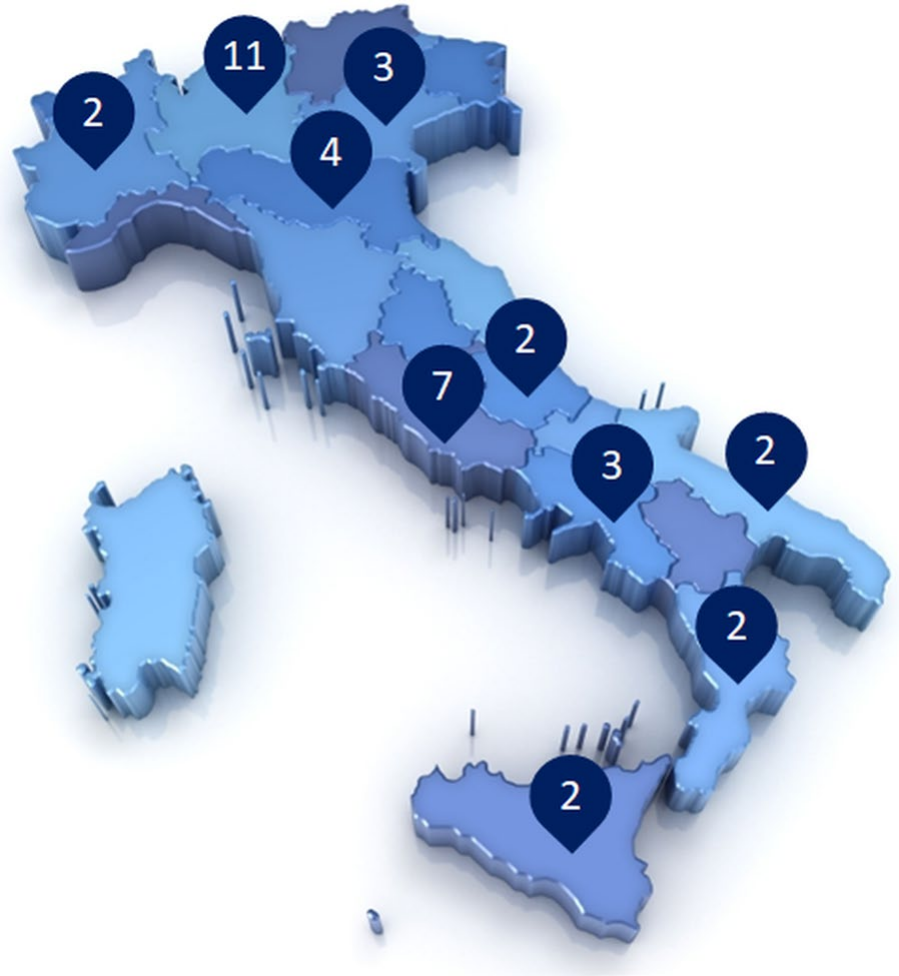
Geographic distribution of respondents

In the first round of the Delphi survey, there were 40 respondents out of 42 invited in the expert group (95%), equally divided according to the two specialties. Thirty-two (80%) of the respondents were males and eight (20%) females, with a nationwide homogeneous distribution (52.5% from the North-Centre of Italy, 47.5% from South-Centre of Italy) (Fig. 2)

The clinical experience and the professional role of the respondents is detailed in Table 2, with 88% of them working for > 25 years and 60% of them > 30 years. The panel represented 32 national and international Scientific Societies.

**Table 2:** Clinical experience and professional role of the respondents

Characteristic	Frequency (n = 40)
Clinical experience (years)	
29	1 (2.5%)
45	4 (10.0%)
56	11 (27.5%)
66	9 (22.5%)
76	10 (25.0%)
> 40	5 (12.5%)
Unit director	39 (97.5%)
Academic role	
Full professor	19 (47.5%)
Associate professor	8 (20.0%)
Professor on contract	5 (12.5%)
PhD	1 (2.5%)

In round 1, a positive consensus was reached for 21/21 statements (100%). Table 3 summarizes the statements and presents the percentage of agreement/disagreement for each one based on the responses of the 40 panellists. Major statements, grouped for macro-areas, are reported below.

**Table 3:**
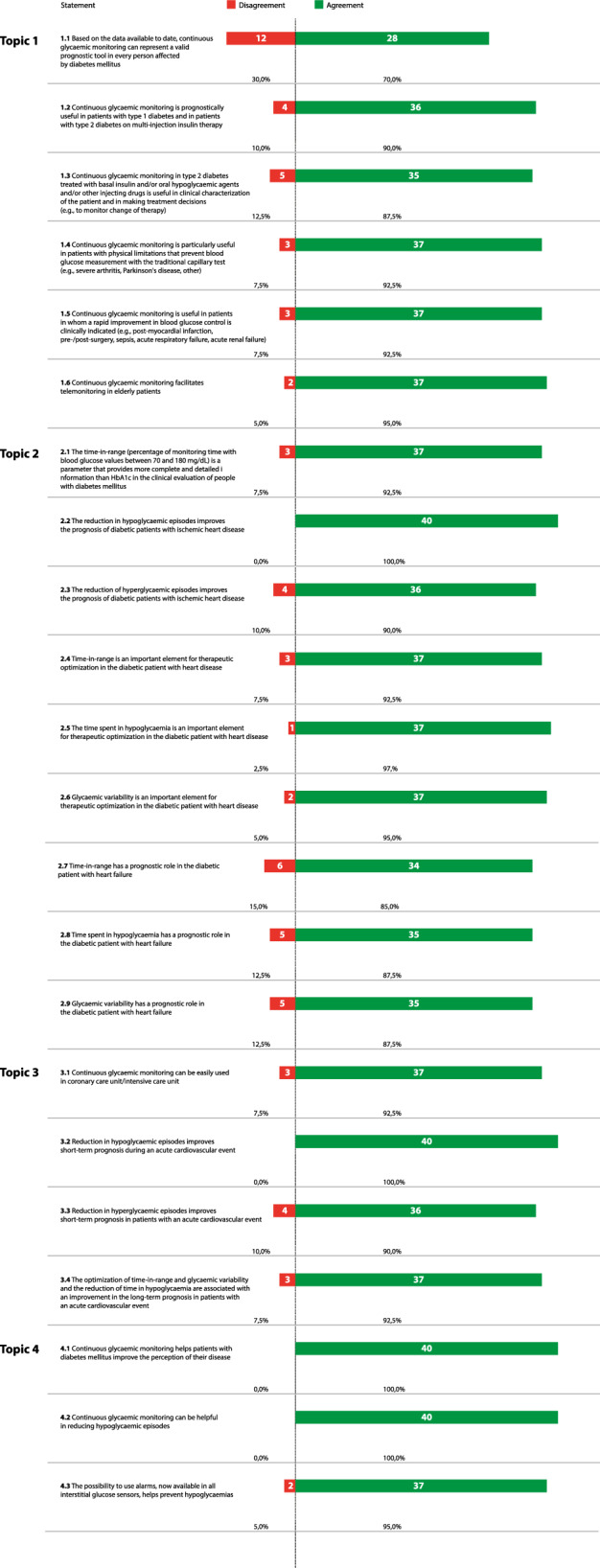
Level of agreement for each statement

### Type of patients who can benefit from continuous glucose monitoring (*FGM *and rt*CGM*)

The experts strongly agreed that CGM is a prognostic tool for T1DM and T2DM treated with multiple daily insulin injections (90%), and, to a lesser extent, for every diabetic patient (70%). Moreover, they firmly believe that CGM is clinically useful to better characterize patients independently from the anti-hyperglycaemic agent(s) they are taking (88%), for patients with physical limitations preventing the correct use of the capillary test (93%), in the acute setting (93%), and as a valid tool for remote monitoring for elderly subjects (95%). The benefit of CGM system in patients with physical limitation preventing measurement of capillary blood glucose is limited to those system which do not require confirmation of estimated glucose by blood testing for clinical decision.

### Prognostic impact of continuous glucose monitoring in diabetic patient affected by heart disease

A full positive consensus was reached considering that the reduction of hypoglycaemic episodes improves the prognosis in patients with ischaemic heart disease (100%), but also hyperglycaemic episodes are similarly perceived (90%). The experts strongly retain that TIR provides more complete and detailed information than HbA1c in the clinical evaluation of people with diabetes (93%), and that it represents a useful tool for optimization of treatment in those with heart disease (93%). The consensus, even to a greater extent, was also obtained for the time spent in hypoglycaemia (98%) and for GV (95%) in diabetic patients with heart disease.

A positive, but less strong, consensus was also reached in the field of heart failure (HF) as regards the prognostic role of TIR (85%), time spent in hypoglycaemia (88%) and GV (88%).

### CGM use during acute cardiovascular events

According to the expert panel, CGM can be easily used in coronary care unit or intensive care unit (93%). In the setting of acute CV events, the experts fully agreed that the reduction of hypoglycaemic episodes improves the short-term prognosis (100%) as well as for the reduction of hyperglycaemic episodes (90%). In addition, they also agreed that the optimization of TIR and GV and the reduction of the time spent in hypoglycaemia can improve the long-term prognosis (93%).

### Insulin/hypoglycaemia education

A full positive consensus was obtained in considering CGM helpful for patients with diabetes to improve the perception of the disease (100%) and to reduce the hypoglycaemic episodes (100%), also thanks to the possibility to set alarms to prevent them (95%).

## Discussion

CGM is currently recommended and reimbursed by the Italian National Health Service for the management of patients with T1DM and T2DM treated with multiple daily insulin injections [16]. However, a growing need is felt by Italian experts to extend its use to patients with T2DM, regardless diabetes treatment, with special needs or at high risk of complications. Indeed, quantifying the duration and magnitude of glycaemic excursions provides another means of assessing glucose control, which is perceived by the panel as complementary and more complete than SMBG and HbA1c [10]. Blood glucose is a vital parameter that in physiology changes in relation to meals, physical activity and all conditions that generate stress; in pathological conditions, such as in people with diabetes, glycaemia also changes with therapies, particularly those that can lead to severe reductions such as insulin. GV is a process characterized by amplitude, frequency, and duration of the fluctuations. Consequently, GV is directly related to hypoglycaemic and hyperglycaemic episodes, as well as their duration and TIR [21]. It is well known that GV is an independent risk factor for diabetes complications, including cardiovascular diseases, acting through the oxidative stress pathway [22–26], and has effects on cognitive function and quality of life [27]. Moreover, increased GV is strongly associated with mortality in the intensive care setting [28, 29]. Furthermore, the objective of diabetes control is to keep blood glucose levels into an accepted range, since deviations from the range in both directions are harmful, increasing the risk of complications with the amplitude of the deviations. Two large randomized controlled trials have demonstrated a significant reduction of hypoglycaemic events and GV, as well as increase in TIR and patients’ satisfaction in subjects with T1DM [30] and insulin treated T2DM [31] managed with FGM, as compared to SMBG. In addition, real-world studies showed greater reductions in HbA1c levels using FGM compared to SMBG [32, 33], also showing that the number of glucose scans is inversely associated with time spent in hypoglycaemia or hyperglycaemia and is positively correlated with TIR [34]. Consequently, besides hypoglycaemic, and hyperglycaemic episodes, according to the panel, also GV and TIR should now be assessed as part of the routine management of diabetes [10]. In addition, the availability of monitoring data in a simple and standardized format, such as the Ambulatory Glucose Profile [11], can facilitate their use in routine clinical practice, enhancing treatment adjustments and improving patient education. In this regard, given the evidence reported above, the experts believed that CGM can have a prognostic role in patients for whom it is recommended by guidelines and reimbursed by the Italian NHS, but also to other to whom the recommendation may be extended.

Diabetes is a traditional risk factor for CV disease, carrying a higher risk for sudden cardiac death, accelerated atherosclerosis, ischaemic heart disease, cardiomyopathy, and HF [35]. In addition, oscillating glucose is considered to have more deleterious effects than constant high glucose levels on endothelial function [36] and postprandial glycaemic spikes may be a more robust determinant of CV disease risk than average glucose levels [37]. This is explained by the hyperglycaemia-induced activation of oxidative stress pathways and inflammation as well as by the rapid formation of advanced glycosylated end-products (AGEs) [38]. However, a doubtful reduction of coronary artery disease by glucose-lowering treatment was found in large meta-analyses [39, 40], possibly because the benefits were partly counterweighed by an increased occurrence of severe hypoglycaemic episodes, associated with the intensive insulin therapy. In fact, hypoglycaemia can be associated with the development of adverse CV outcomes by means of several mechanisms, including blood coagulation abnormalities, inflammation, endothelial dysfunction, and sympathetic responses [35, 41]. Accordingly, the panel agreed that CGM with the new glucometrics may have a prognostic role in patients with ischaemic heart disease. Notably, although to a lesser extent than hypoglycaemia, also hyperglycaemic episodes are perceived as prognostically deleterious by the panel.

Diabetes is also a risk factor for HF [42] and left ventricular dysfunction can be found in up to 40% of diabetic people [43]. The link between the two conditions goes beyond ischemic heart disease, but also involves several other mechanisms, including micro-circulatory dysfunction, metabolic derangements with lipotoxicity, cytokine and renin–angiotensin–aldosterone system activation, altered calcium handling, and endothelial dysfunction [44, 45]. Oxidative stress and inflammation seem to be at the basis of the phenomenon and are also the hallmark of HF with preserved ejection fraction, as currently hypothesized [46, 47]. Finally, also diabetic neuropathy and consequently cardiac autonym dysfunction can play a role in this context [48]. The interest about the link between HF and diabetes is recently growing in the cardiologic scenario after the publication of SGLT2 inhibitors trials, which demonstrated beneficial effects of these drugs in HF patients, which was in fact independent from diabetes itself [49, 50]. The panel agreed in considering hypoglycaemic events, TIR and GV as having a prognostic role in HF patients with diabetes, even if dedicated studies are still lacking. This perception should prompt future research in this field.

Mortality in acute myocardial infarction and other acute CV events is also increased in patients with diabetes [51, 52]. This may occur independently from the extent of myocardial infarct size, because of various negative effects, including increased inflammation, endothelial dysfunction, pro-thrombotic state and oxidative stress [52]. Acute hyperglycaemia characterizes up to 50% of patients admitted for myocardial infarction [53], and for every 18 mg/dL (1 mmol/L) increase in glucose level above 200 mg/dL, it has been reported a 4% and a 5% increase in hospital mortality risk in patients without and with diabetes, respectively [54]. In addition, impaired control of glycemia in this setting has been associated with severe coronary flow impairment, increased left ventricular dysfunction, larger infarct size, and higher risk of acute HF, cardiogenic shock, and acute kidney injury [53]. On the other hand, also acute myocardial infarction patients with hypoglycaemia appear to have worse outcomes [55, 56], including myocardial ischaemia and arrhythmias [57]. Furthermore, both hypoglycaemia and hyperglycaemia are associated with a three-fold increased risk of 30-day mortality when compared with euglycaemic patients, thus determining a U-shaped relationship between blood glucose levels and adverse outcomes [56]. Consequently, the consensus is that both hyperglycaemia and hypoglycaemia should be avoided in critically ill patients [58]. In fact, acute variability of glucose values negatively correlates with the proportion of reversibly injured myocardial tissue that does not progress to infarction [59]. GV > 49 mg/dL was demonstrated to be the strongest independent predictor of mid-term major adverse cardiac events (death for cardiac cause, new-onset myocardial infarction, acute HF) [60]. Finally, the experts agreed that the CGM can be useful in intensive care unit as it carries prognostic information at short-term (e.g., in-hospital mortality). In addition, the improvement in TIR, GV and time spent in hypoglycaemia is perceived to also improve the long-term prognosis of an acute CV event, even if specific data are lacking.

CGM use was demonstrated to improve patients’ quality of life, with a greater satisfaction than SMBG [30, 31, 61]. In fact, according to the expert panel, the system has an educational aspect, helping patients’ perception of their own disease and increasing the adherence and the confidence to treatment. Notably, CGM reduces hypoglycaemic episodes, which are most feared therapy complication [30, 31], particularly by the activation of dedicated alarms in the more recent systems, which can help preventing them.

## Conclusions

The results of this Delphi survey suggest that the use of CGM systems may have an important clinical and prognostic role in patients with diabetes beyond the current recommendations. In particular, the wealth of data provided by CGM devices, the availability of new glucometrics, patients’ satisfaction, the suggested improvement of clinical outcomes and the possibility of remote monitoring, thanks to in cloud platforms, are key elements favouring CGM use in patient at risk or with overt CV disease, particularly in those with ischaemic heart disease and HF, both in the chronic and acute setting. Dedicated studies are needed, however, to confirm the experts’ feelings.

## Data Availability

The dataset supporting the conclusions of this article is available and included within the article.

## Abbreviations

CGM: Continuous glucose monitoring
TIR: Time in range
GV: Glycaemic variability
HbA1c: Glycated haemoglobin A1c
SMBG: Self-monitoring blood glucose
ADA: American Diabetes Association
T1DM: Type 1 diabetes mellitus
T2DM: Type 2 diabetes mellitus
CV: Cardiovascular
CVOT: Cardiovascular outcome trial
KOL: Key opinion leader
HF: Heart failure
